# Effectiveness of music therapy for children with autism spectrum disorder: meta-analysis and potential biological mechanisms

**DOI:** 10.3389/fpsyt.2025.1722874

**Published:** 2026-01-30

**Authors:** Li Wu, Xinming Zhang, Shuyue Liu, Xinping Gao, Jun Wang

**Affiliations:** 1 School of Music and Dance, South-Central Minzu University, Wuhan, Hubei, China; 2 National 111 Center for Cellular Regulation and Molecular Pharmaceutics, Hubei University of Technology, Wuhan, Hubei, China; 3 International Center for Redox Biology and Precision Medicine of Hubei Province, Hubei University of Technology, Wuhan, Hubei, China; 4 Hubei University of Technology Autism and Depression Diagnosis and Intervention Institute, Hubei University of Technology, Wuhan, Hubei, China; 5 Ningbo Symphony Orchestra, Ningbo, Zhejiang, China

**Keywords:** autism spectrum disorder, children, mechanism, meta-analysis, music therapy

## Abstract

**Objectives:**

Autism spectrum disorder (ASD) is a complex neurodevelopmental disorder characterized by deficits in social interaction, communication skills, and repetitive behaviors. However, no effective pharmacological treatments targeting core symptoms have yet been developed. As a non-pharmacological intervention, music therapy (MT) is increasingly being explored for its potential value in improving functional outcomes for children with autism. This study aims to examine the efficacy of MT for children with ASD through a meta-analysis.

**Method:**

We conducted a systematic review following the PRISMA guidelines. A comprehensive and systematic search of PubMed, Web of Science, and the Cochrane Library databases up to August 2025 was performed to identify studies on MT treatment for children with ASD. Continuous variables were reported as standardized mean differences (SMD) with 95% confidence intervals (CI). All analyses were conducted using Stata statistical software version 17.0.

**Results:**

Our meta-analysis included 18 studies. Results showed that MT significantly reduced the total score on the Autism Behavior Checklist (ABC) (SMD = -0.76, 95% CI: -1.31 to -0.22, P = 0.01) and Childhood Autism Rating Scale (CARS) total scores (SMD = -0.43, 95% CI: -0.73 to -0.14, P < 0.01). Specifically, compared with the control group, MT significantly improved social skills (SMD = -0.26, 95% CI: -0.46 to -0.05, P = 0.01), behavioral abilities (SMD = -0.72, 95% CI: -1.06 to -0.39, P < 0.01), and sensory (SMD = -0.87, 95% CI: -1.68 to -0.07, P = 0.03), emotional (SMD = -1.31, 95% CI: -1.98 to -0.64, P < 0.01), and verbal functioning (SMD = -0.65, 95% CI: -0.19 to 1.12, P = 0.01).

**Conclusion:**

In summary, MT demonstrates efficacy in improving behavioral symptoms associated with autism spectrum disorders. However, caution should be exercised when interpreting results due to limited research literature in some analyses. Further studies are needed to validate its therapeutic effects.

**Systematic review registration:**

https://www.crd.york.ac.uk/prospero/, identifier CRD420251252228.

## 1 Introduction

Autism spectrum disorder (ASD), also known as autism, is a complex neuro-developmental condition characterized by impairments in behavior, communication, and social functioning to varying degrees (1). Typically onset occurs in early childhood and gradually manifests within the first five years of life, persisting through adolescence and into adulthood. Over the past 50 years, the prevalence of autism has steadily increased worldwide, with males being diagnosed at a rate more than four times higher than females (2). According to the 2020 report from the Centers for Disease Control and Prevention’s Autism and Developmental Disabilities Monitoring (ADDM) Network, approximately 1 in 36 children is diagnosed with autism (CDC). In recent years, this prevalence has continued to rise, with a global prevalence rate of about 7.6 ‰ (3).

The etiology of autism remains under investigation, but various interventions are currently available to alleviate symptoms, reduce the burden on individuals and their families, and enhance their life skills and overall well-being. As autism is a developmental disorder, early intervention is crucial for improving symptoms, learning, and development (4). Currently, there is a lack of specific medications to alleviate the core symptoms of individuals with ASD (5). Risperidone and aripiprazole are only approved by the U.S. Food and Drug Administration (FDA) for controlling irritability associated with the disorder. The National Institute for Health and Care Excellence (NICE) recommends three potential interventions: psychosocial interventions targeting core symptoms, psychosocial interventions focusing on life skills, and biomedical interventions. The prognosis for children with autism has historically been poor, with only 25% demonstrating “good” or “fair” outcomes. Similar rates are observed among adults with autism. Most adults with autism remain dependent on family or professional care. They continue to face challenges in accessing formal education, maintaining employment, living independently, and sustaining interpersonal relationships (6). Therefore, there is a need for therapeutic, improved treatment approaches and extensive research to investigate the effectiveness of current interventions. In autism treatment, alternative and non-pharmacological interventions utilizing auditory and sensory integration practices are gaining increasing attention. Research indicates that MT holds promising application prospects, with some studies further confirming its efficacy as a behavioral intervention (7, 8).

MT is a clinical practice that delivers evidence-based interventions within a therapeutic framework. Certified professionals utilize evidence-supported musical interventions within a therapeutic relationship to achieve individualized goals. It can be applied to promote health, alleviate stress, reduce pain, facilitate emotional expression, enhance memory, improve communication, and support physical rehabilitation—among other medical and educational objectives. Group music therapy offers an indirect form of communication that enhances engagement among individuals with autism. By observing the social behaviors of autistic children, significant progress has been noted in areas such as joint attention, eye contact, and turn-taking interactions. Consequently, group MT aims to build relationships through music, encouraging patients to establish social connections with others.

The purpose of MT is to develop cognitive, motor, emotional, social, sensory, and learning skills, and to transfer skills developed through music to other areas of life (9). During receptive and active music-making experiences, both the superior temporal lobe and inferior frontal lobe are activated. Enhanced synchrony between these regions promotes collaborative functioning among cognitive, sensorimotor, and perception-action regulation areas, thereby strengthening sensory integration capabilities (10). Research has found that individuals who engaged in long-term music listening exhibited the most significant changes in the volume and density of their cerebral cortex and cerebellum, autistic children who participated in sustained musical experiences demonstrated larger corpus callosum, frontal lobe, temporal lobe, and motor areas (11, 12). Research indicated that children participating in music intervention exhibited increased functional connectivity between bilateral primary auditory cortex, subcortical regions, and motor areas compared to those receiving non-music interventions. Against the backdrop of rising autism prevalence, there is a growing need to develop interventions that offer new insights and perspectives, while also addressing the particularly high demand for services among adolescents and young adults with autism (10).

In this review article, we systematically reviewed relevant studies and employed meta-analysis methods to summarize the therapeutic effects of MT on children with ASD. Compared to previous related analytical articles, we have further explored in detail the potential mechanisms of MT, along with its future therapeutic strategies and challenges.

## 2 Materials and methods

This systematic review adheres to the Preferred Reporting Items for Systematic Reviews and Meta-Analyses (PRISMA) guidelines for reporting and has been prospectively registered in the PROSPERO database under registration number CRD420251252228.

As of August 30, 2025, searches were conducted in PubMed, the Cochrane Library, and Web of Science. An example of a PubMed search is: (music therapy) OR (Therapy, Music)) AND (Autism Spectrum Disorder, Autistic OR Disorders, Autistic OR Kanner’s Syndrome OR Kanner Syndrome OR Kanner3 Syndrome OR Autism, Infantile OR Infantile Autism OR Autism OR Autism, Early Infantile OR Early Infantile Autism OR Infantile Autism).

For each search, titles and abstracts were screened, and full-text versions of articles meeting the criteria were downloaded. The full texts were reviewed, and any reference articles not yet obtained were ordered and acquired.

### 2.1 Inclusion and exclusion criteria

Studies included in the meta-analysis should meet the following criteria (1): Participants were diagnosed with ASD according to internationally recognized diagnostic criteria, such as the Diagnostic and Statistical Manual of Mental Disorders (DSM-IV or V) or the International Classification of Diseases, 10th Revision (ICD-10) classification of mental and behavioral disorders; (2) Studies reported research on MT for patients with ASD; (3) These studies employed music within experimental or observational research designs; (4) Studies reporting measurable outcomes or results were included.

Exclusion criteria include: (1) Receiving standalone MT within 6 months prior to the study; (2) Exclusion of hospital reports, review articles, qualitative studies, and case reports if the study was a systematic review; (3) Exclusion of studies unrelated to ASD, or those involving patients with hearing impairment or other complications; (4) Exclusion of studies involving non-music-related interventions; (5) Exclusion of studies lacking a clearly defined control group, incomplete data, or insufficient documentation.

### 2.2 Data extraction

Authors W, Z, and L independently searched the literature, screened titles and abstracts, and downloaded articles for inclusion. Decisions regarding article inclusion were made by consensus, with final decisions made by author W when necessary.

The quality of included randomized controlled trials was assessed using the Cochrane Risk of Bias Tool. This tool evaluates risk of bias across six domains: selection bias (including random sequence generation and allocation concealment), implementation bias (including blinding of investigators and participants), measurement bias (blinded assessment of study outcomes), follow-up bias (completeness of outcome data), reporting bias (selective reporting of study outcomes), and other biases (additional sources of bias), totaling seven items.

### 2.3 Statistical analysis

All studies meeting the criteria were extracted using standardized data collection forms. Mean values and standard deviations before and after MT were manually entered into Stata for analysis. The null hypothesis of this study was that MT improves behavioral characteristics in children with ASD. Meta-analysis was conducted using Stata software (version 17.0). A random-effects model was employed to calculate standardized mean differences and 95% confidence intervals, with the I² statistic used to assess statistical heterogeneity among studies. When the p-value of the Z-test was ≤ 0.05, the pooled results from multiple studies were considered statistically significant. Effect sizes were interpreted using SMDs. If heterogeneity was present, sensitivity analyses were conducted by sequentially removing individual studies to identify sources of variation. Throughout this review, we downloaded and adhered to the Preferred Reporting Items for Systematic Reviews and Meta-Analyses (PRISMA) guidelines.

## 3 Result

### 3.1 General results of the included studies

**Figure 1:**
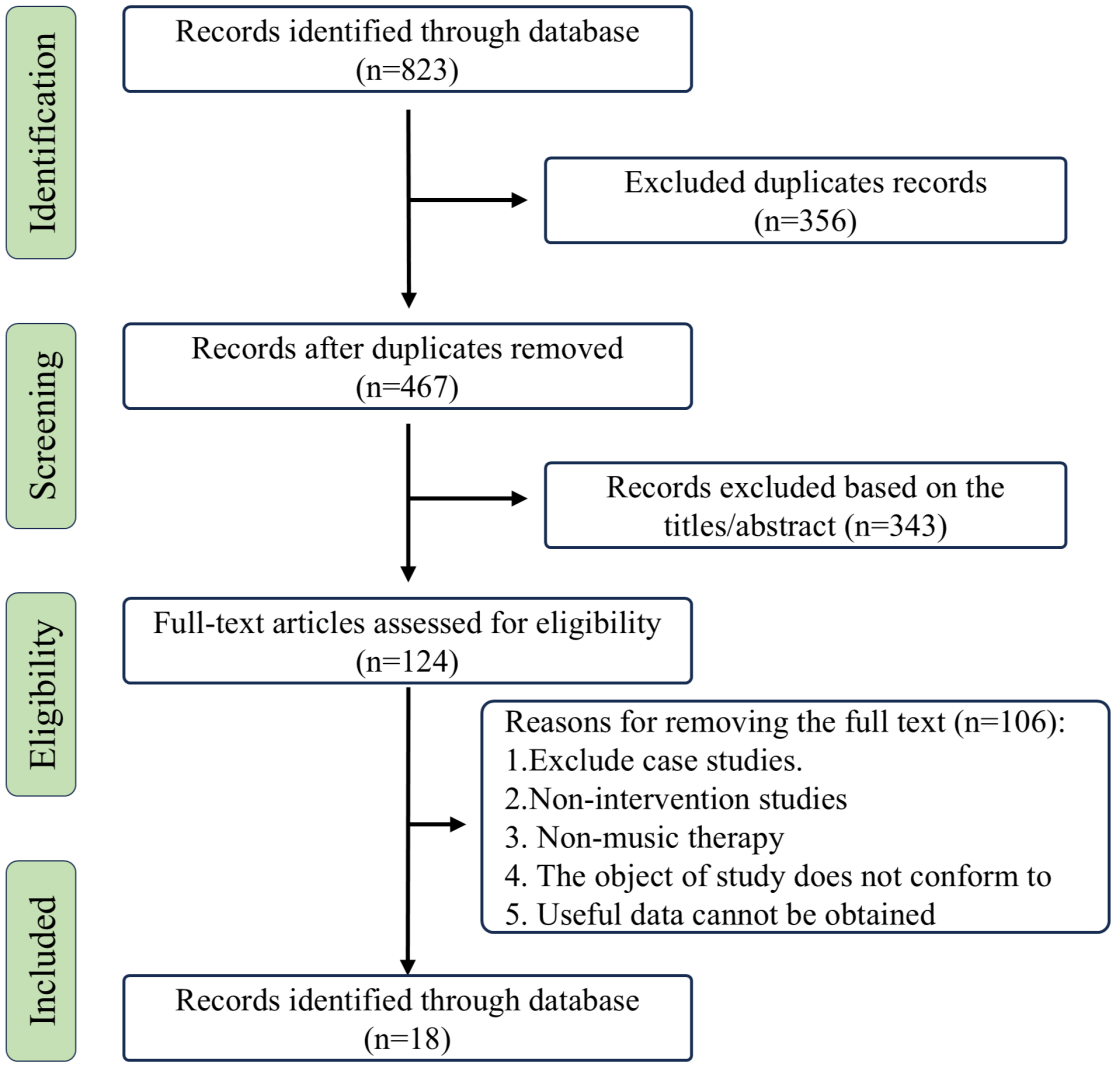
PRISMA flow chart. Showing the article selection process.

A single inclusion criterion was applied, limiting selection to research articles only. By August 2025, 292 studies were retrieved from PubMed, 102 from the Cochrane Library, and 429 from Web of Science, totaling 823 studies. After removing duplicates and screening titles, abstracts, and full texts, 124 studies were obtained. Of these, 106 were excluded as irrelevant to our meta-analysis. The primary objective of this study was to investigate improvements in multiple behavioral abilities among children with ASD following MT compared to standard treatment or “placebo” treatment. **Figure 1** illustrates the overall search strategy and article screening process, while **Table 1** presents the basic information and study characteristics of MT participants, along with the overall treatment effects for each study. The music styles employed in the experimental groups varied widely, including children’s songs, original compositions created by therapists, and other musical genres. Across the included studies, MT sessions lasted from 3 days to 9 months.

**Table 1:** Characteristics of included studies.

Study	Sample size		Age (year)		Music genre	Duration
	MT (males/females)	Ctrl (males/females)	MT	Ctrl		
Buday, Evelyn M (13)	10 8/2	10 8/2	4.25~9		Goldilocks Returns	2 weeks
Corbett (1)	5 7/1	6 5/1	5.46 ± 3.12		Tomatis sound	36 weeks
Zhang (14)	41 36/5	40 34/6	5.46 ± 3.12	4.84 ± 3.02	unclear	24 weeks
Wang J (15)	26	26	4.2 ± 5.68		Nursery rhyme	6 weeks
Hayoung A Lim (16)	11	11	3-5		unclear	2 weeks
Chen (17)	15	15	2~6		Improvisational music	12 weeks
Hayoung A Lim (18)	18	14	3~5		unclear	3 days
Gustavo Gattino (19)	12	12	7~12		unclear	20 weeks
Edward, T. Schwartzberg (20)	7	4	9~21		Music based on social stories	1 week
Chen (21)	9	9	4~5		Music based on social stories	12 weeks
G. A. Thompaon (22)	11	10	3.66 ± 0.53	3.91 ± 0.56	Family-centred music	16 weeks
Ghasemtabar SN (23)	13 7/6	14 7/7	8.96 ± 1.36	9.23 ± 1.54	Orff music	45 days
Sam Porter (24)	16	18	8~16		unclear	12 weeks
Latif, N (25)	19 15/4	15 13/2	9.5~12.9	8.13~11.9	Playing musical instruments	12 weeks
M. EI Tellawy (26)	38	25	5.9 2 ± 2.1	7.2 ± 2.92	Tomatis sound	12 weeks
He YS (3)	30 25/5	30 22/8	4.7 ± 1.2	5.1 ± 1.3	Mozart’s Music and Orff Music	8 weeks
Fan QL (27)	48 35/13	45 30/15	4.51 ± 0.88	4.56 ± 0.93	Orff music	24 weeks
Zhou ZW (28)	15 9/6	14 9/5	4.67 ± 1.39	4.53 ± 1.31	Chinese nursery rhymes and Orff music	12 weeks

### 3.2 Risk of bias included in the study

**Figure 2:**
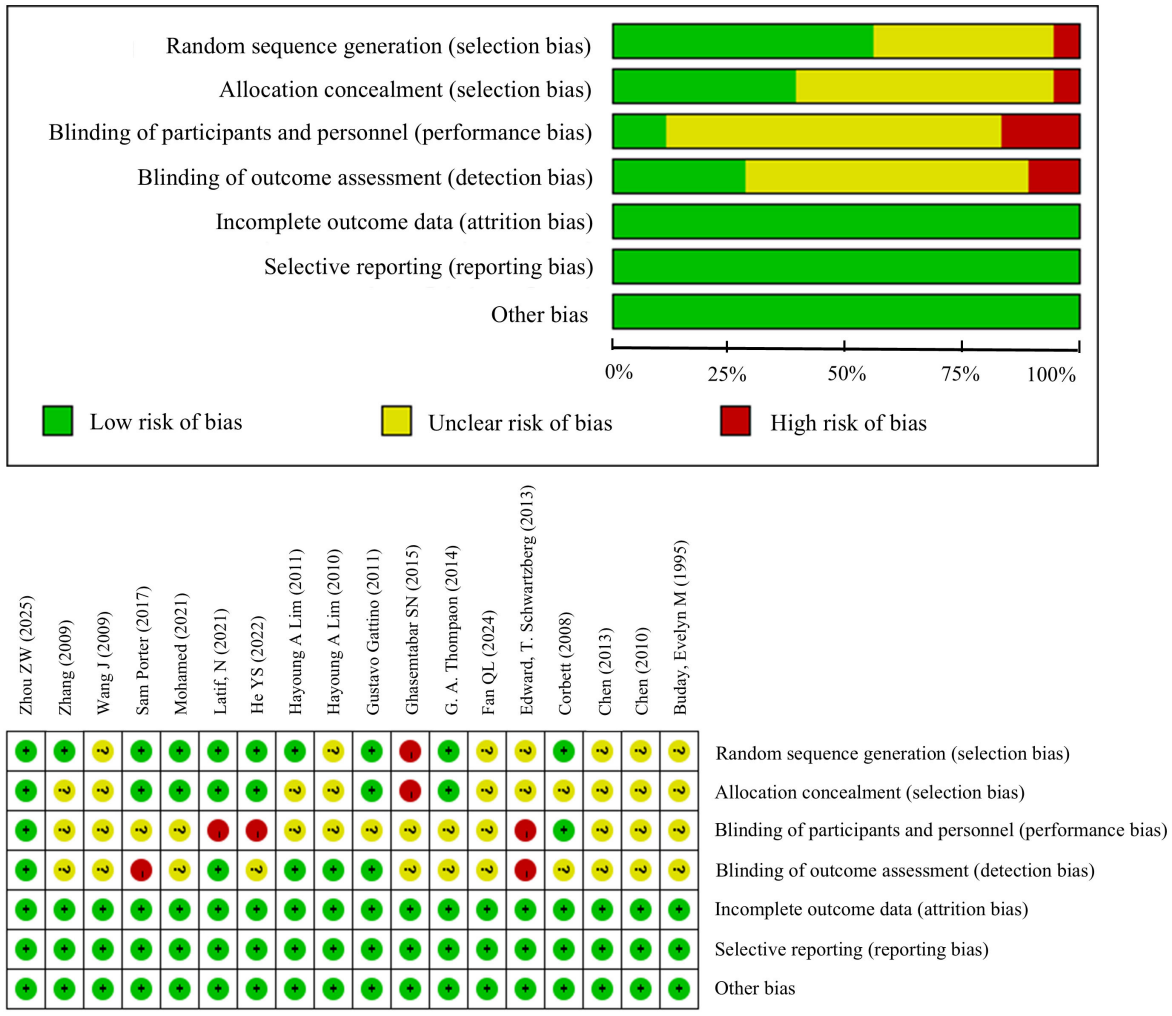
Risk of bias graph: review authors’ judgements about each risk of bias item presented as percentages across all included studies.

The risk of bias in the included studies was assessed using the Cochrane Risk of Bias Assessment Tool. **Figure 2** summarizes the overall risk of bias across included studies. Ten trials of randomized groups using computer-generated randomization lists. Seven of these studies reported allocation concealment. Five studies described blinded assessment of outcome measures. All included studies were randomized controlled trials providing high-quality evidence.

### 3.3 The impact of MT on the total ABC score and CARS score

The ABC was developed by Krug in 1978 (29), The ABC Scale lists 57 behavioral characteristics of children with autism, covering five domains: Sensory, Relating, Body and Object Use, Language, and Social and Self-help. It is designed for children aged 2 to 14 and is rated by parents or teachers. The total score is 158 points, with a screening cutoff score ranging from 53 to 67 points. The CARS, developed by Schopler et al., is designed for children aged 2 years and above (30). It consists of 15 items rated on a Likert 4-point scale, with total score of 60 points. A total score of 30–36 indicates mild to moderate autism, while a score ≥ 37 is classified as severe autism.

**Figure 3:**
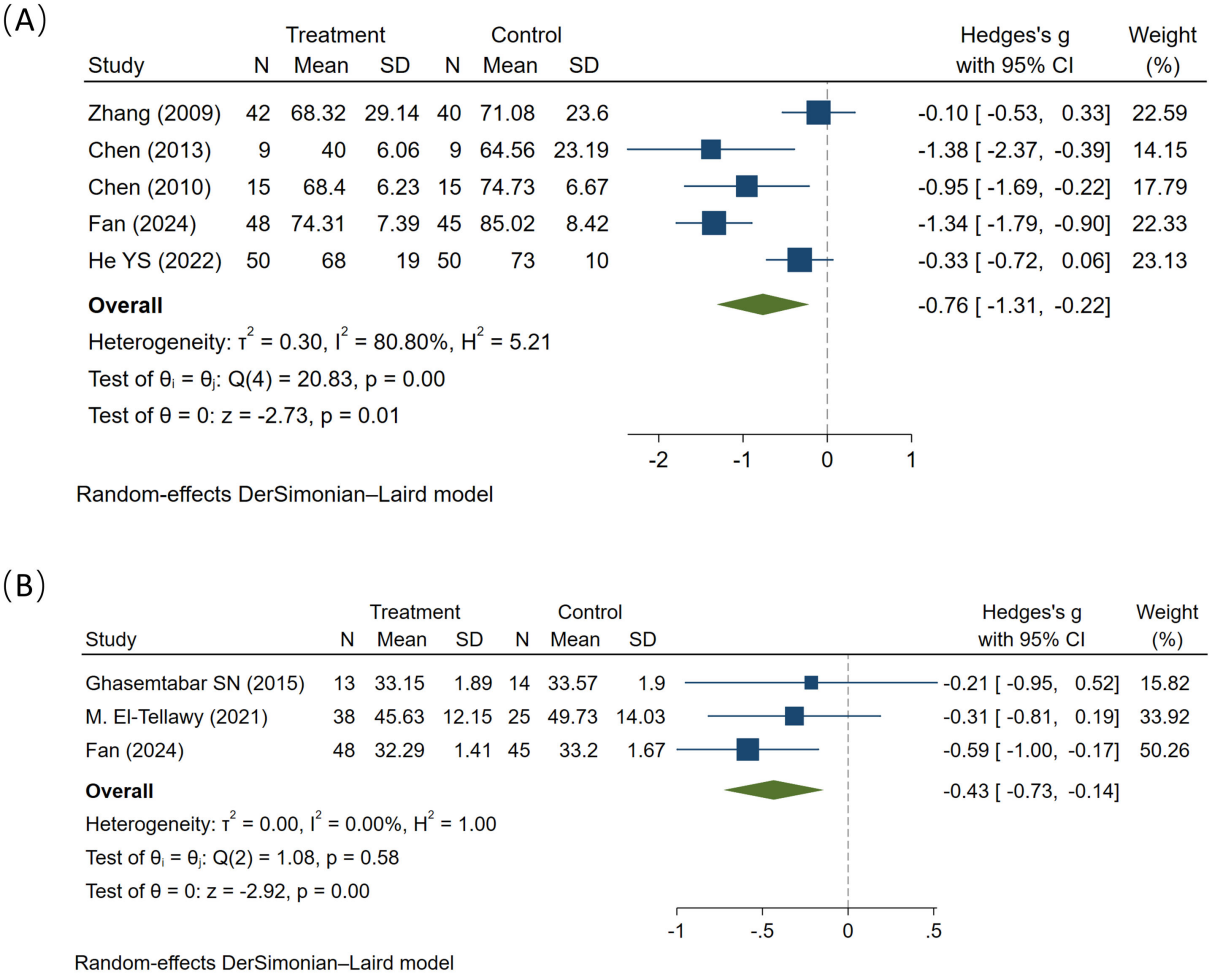
The effect of music therapy on total ABC scores **(A)**, and total CARS score **(B)**.

Five studies reported the effects of MT on the total ABC scores, involving a total of 321 participants (164 in the experimental group and 159 in the control group). Compared to the control group, MT significantly reduced the total ABC scores (SMD = -0.76, 95% CI: -1.31 to -0.22, I² = 80.8%, P = 0.01) (**Figure 3A**), with high heterogeneity observed among the studies, Subgroup analysis by intervention duration explored sources of heterogeneity. MT significantly reduced ABC scores in the >12-week subgroup (I² = 93.5%, P < 0.01), but had no significant effect on scores in the ≤ 12-week subgroup (I² = 61.8%, P = 0.073) (**Supplementary Figure S1A**). The funnel plot asymmetry did not indicate significant publication bias. Sensitivity analyses confirmed the robustness of the directional conclusions, though effect sizes were influenced by individual studies (**Supplementary Figures S1B, C**). Overall, the results indicate that music therapy effectively improves the severity of symptoms in children with autism and demonstrates significant advantages over control condition treatments. This improvement becomes more pronounced with longer duration of music therapy.

Three studies reported the effects of MT on the total CARS scores, involving a total of 183 participants (99 in the experimental group and 84 in the control group). Compared to the control group, MT significantly reduced the total CARS scores (SMD = -0.43, 95% CI: -0.73 to -0.14, I² = 0%, P < 0.01), with no heterogeneity observed among the studies (**Figure 3B**). Overall, the results indicate that MT effectively improves the severity of symptoms in children with ASD and demonstrates significant advantages compared to control condition therapy.

### 3.4 The impact of MT on social functioning

Children with ASD commonly experience impairments in social interactions with their peers, which significantly affects their own learning and daily life, as well as that of their families (31).

**Figure 4:**
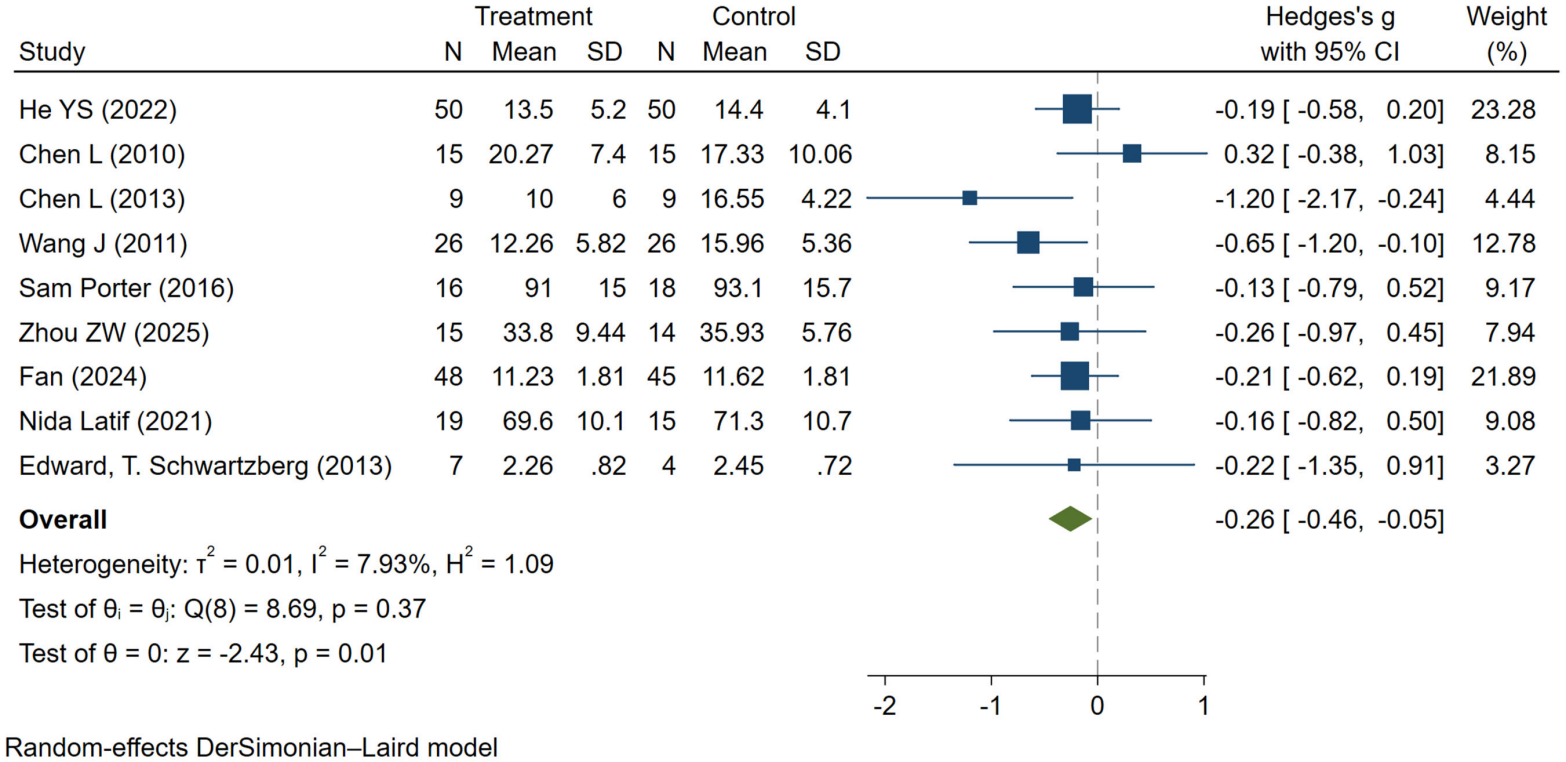
The effect of music therapy on social functioning.

Nine studies provided specific scores regarding the impact of MT on the social functioning of individuals with autism, involving a total of 401 participants (205 in the experimental group and 196 in the control group). Compared to the control group, MT significantly reduced social impairment scores (SMD = -0.26, 95% CI: -0.46 to -0.05, I² = 7.93%, P = 0.01), with low heterogeneity observed across studies (**Figure 4**). A comprehensive analysis indicates that MT leads to statistically significant improvements in social functioning among children with ASD, enhancing their social abilities and demonstrating advantages over control condition therapy. The results are robust through sensitivity analysis. Funnel plot asymmetry did not reveal any potential for publication bias (**Supplementary Figures S2C, D**).

Other studies have similarly found that MT has a profound and lasting impact on improving the social skills of children with autism (23). Additionally, researchers found that family-centered MT not only enhances social interactions within families and communities but also strengthens parent-child relationships (22). MT has a good intervention effect on social interaction, which has also been confirmed in others study (32) and found that music improves brain functional connectivity (33).

### 3.5 The impact of MT on physical behaviors

Abnormal physical behaviors in individuals with ASD are one of the characteristic features that distinguish them from typically developing children (34), A common example is stereotyped behavior, which refers to motor and verbal behaviors that are restricted, repetitive, and serve no apparent adaptive function (35). Researchers have observed that stereotyped behaviors also occur in typically developing children during early childhood. However, children with ASD or other developmental disorders exhibit differences in the duration of vocal and motor stereotyped behaviors. Specifically, older preschool-aged children with ASD or pervasive developmental disorders (PDD) demonstrate longer durations of motor and vocal stereotyped behaviors, while their typically developing peers show lower levels of motor stereotyped behaviors (36).

**Figure 5:**
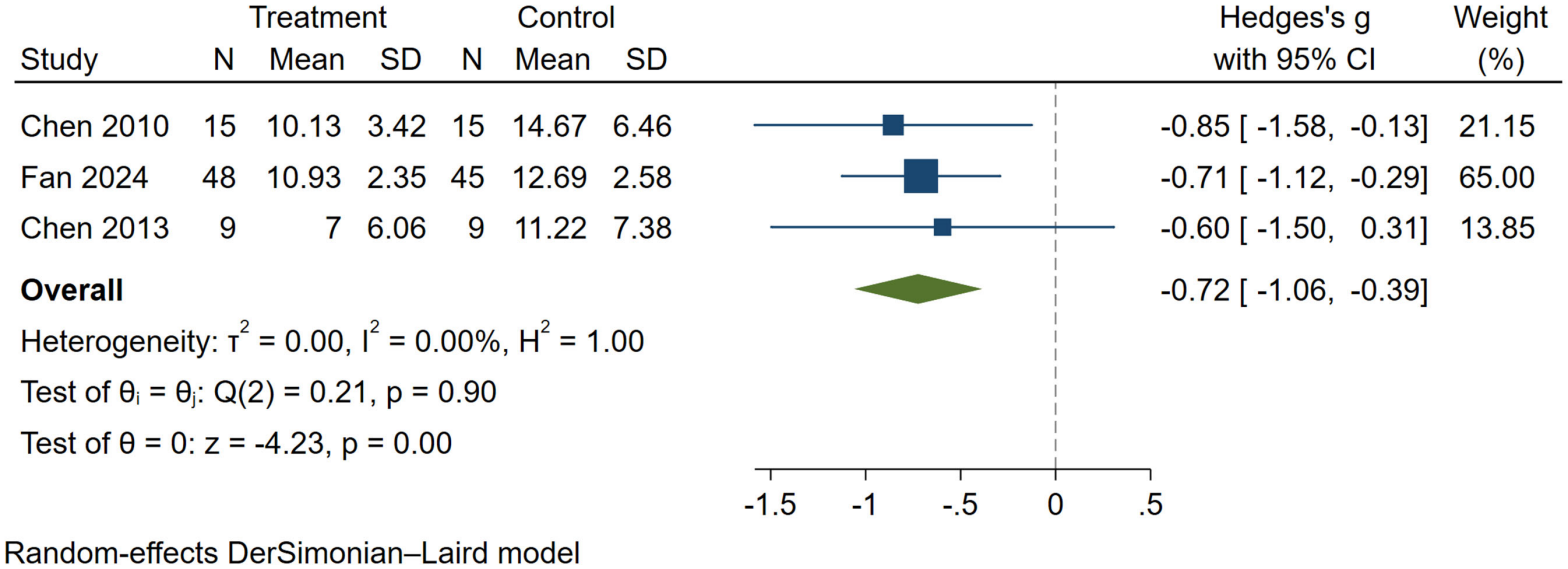
The effect of music therapy on physical behaviors.

In this analysis, three studies comprising a total of 141 participants (72 in the experimental group and 69 in the control group) were included. All studies demonstrated favorable interventional effects of MT on behaviors in individuals with autism. A comprehensive analysis indicated that MT significantly improved sensory processing disturbances in children with ASD (SMD = -0.72, 95% CI: -1.06 to -0.39, I² = 0.0%, P < 0.01) (**Figure 5**), with statistically significant results. The findings suggest that MT exhibits superior efficacy compared to control condition therapy in improving behavioral symptoms in individuals with ASD.

### 3.6 The effect of MT on sensory and emotional scores

One of the core symptoms of ASD is sensory processing dysfunction, which manifests as hyper- or hypo-reactivity to sensory stimuli or unusual interest in sensory aspects of the environment (such as apparent indifference to pain/temperature, adverse response to specific sounds or textures, excessive smelling or touching of objects, visual fascination with lights or movement) (37). These symptoms severely impact patients’ daily functioning.

**Figure 6:**
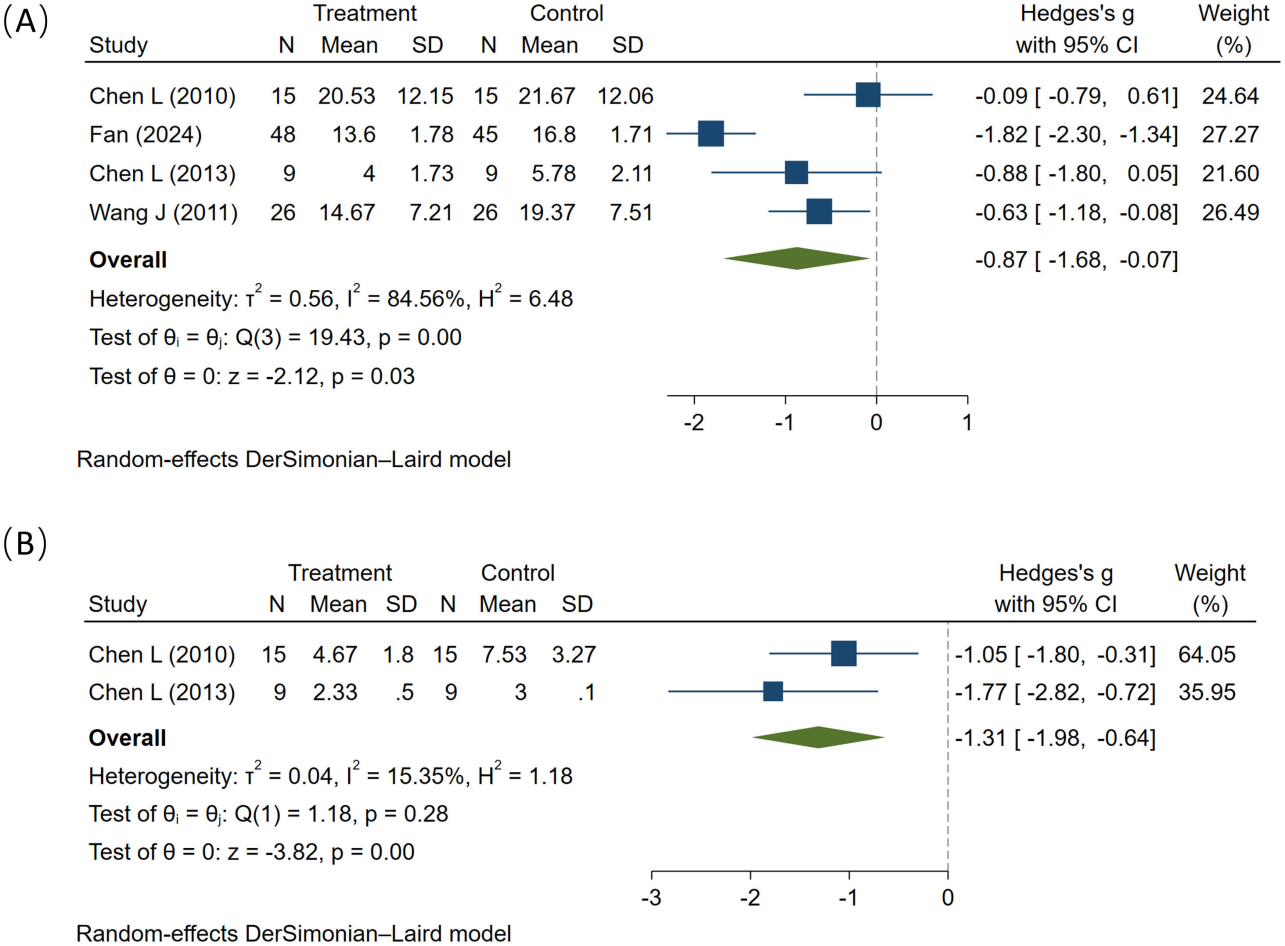
The effect of music therapy on sensory **(A)** and emotional **(B)** scores.

In this analysis, four studies involving a total of 193 participants (98 in the experimental group and 195 in the control group) were included. All studies indicated that MT has beneficial interventional effects on sensory dysfunction. A comprehensive analysis demonstrated that MT significantly improved sensory impairments in children with autism (SMD = -0.87, 95% CI: -1.68 to -0.07, I² = 84.56%, P = 0.03) (**Figure 6A**), Subgroup analysis by intervention duration explored sources of heterogeneity. MT significantly reduced perceived pain scores in the >12-week subgroup (I² = 90.0%, P = 0.002). It reduced perceived pain scores in the ≤ 12-week subgroup (I² = 44.3%, P = 0.18) (**Supplementary Figure S3A**). Due to the limited number of studies included in the analysis, this funnel plot provides little meaningful assessment of publication bias. Furthermore, the results of sensitivity analyses were unstable and highly sensitive to individual studies. (**Supplementary Figures S3B, C**). Overall, compared with the control condition, MT demonstrated more significant therapeutic efficacy in improving sensory dysfunction in ASD patients; however, further high-quality studies are needed to validate these findings.

Regarding the impact of MT on emotions, two studies were included in the analysis, involving a total of 48 participants: 24 in the experimental group and 24 in the control group. Both studies demonstrated that MT improves emotional functioning in children with ASD. A meta-analysis (SMD = -1.31, 95% CI: -1.98 to -0.64, I² = 15.35%, P < 0.01) yielded statistically significant results, indicating that MT demonstrates superiority over control condition in improving the emotions of ASD patients (**Figure 6B**). Similarly, another study found that Tomatis sound therapy can improve the emotional state of children with ASD (26). However, the small sample size of included studies necessitates further experimental evidence.

### 3.7 The impact of MT on verbal and non-verbal scores

The ability to establish connections and communicate with others significantly influences a child’s further development, It also plays a decisive role in establishing social relationships with others. In children with autistic verbal development is very diverse, even if they can speak, they have huge problems with understandingof the meaning of communication and establishing relationships. Lack of social communication with the environment often causes exclusion from the community (38). Therefore, enhancing the verbal abilities of individuals with ASD is one of the primary tasks in improving autistic behaviors.

**Figure 7:**
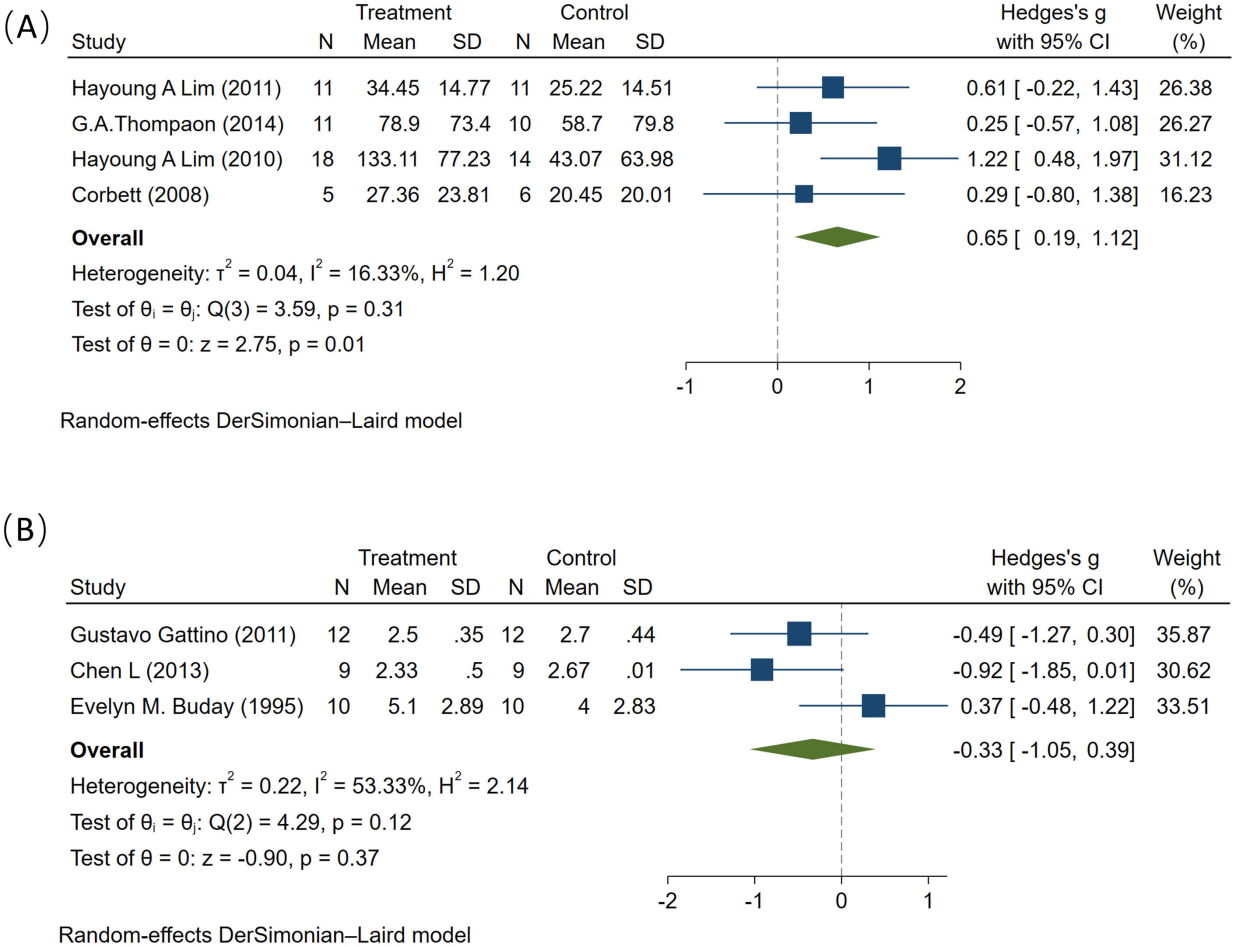
The effect of music therapy on verbal **(A)** and non-verbal **(B)** scores.

In this analysis, four studies examined the impact of MT on verbal scores in ASD patients, involving a total of 86 participants (45 in the experimental group and 42 in the control group). The studies demonstrated low heterogeneity. The pooled analysis showed statistically significant effect of MT on verbal scores (SMD = 0.65, 95% CI: 0.19 to 1.12, I² = 16.3%, P = 0.01) (**Figure 7A**). The results are robust through sensitivity analysis. Funnel plot asymmetry did not reveal any potential for publication bias (**Supplementary Figures S4C, D**).

Additionally, four studies analyzed the impact of MT on non-verbal scores in ASD patients, involving 62 participants (31 in the experimental group and 31 in the control group), with high heterogeneity across studies. The pooled analysis showed no statistically significant effect of MT on non-verbal scores either (SMD = -0.33, 95% CI: -1.05 to 0.39, I² = 53.33%, P = 0.37) (**Figure 7B**).

## 4 Discussion

The purpose of this meta-analysis is to evaluate the efficacy of MT for the general performance, as well as specific function of children with ASD. This is the first time that the effectiveness of MT was evaluated by category. The review of former studies indicated that MT seemed effective in reducing most autism-related symptoms, as assessed by various standardized tools.

This meta-analysis included 18 randomized controlled trials, comprising 344 participants in the MT group and 318 in the CT group, to evaluate the effectiveness of MT as an intervention for individuals with autism. The selected studies analyzed the effects of music intervention on participants’ behavior, verbal, and social skills using the ABC and the CARS standards.

By comparing the MT group and the CT group. Eight studies comprehensively assessed the improvement of autism symptoms in children with ASD through ABC and CARS scores. Overall results indicate that MT represents a more advantageous treatment approach for improving autism symptoms compared to standard therapy. Ten studies investigated social functioning in children with ASD, revealing that MT exerts a positive impact on social interaction. Three studies evaluated physical behavior, with results similarly indicating MT’s beneficial effects on patient behavior. Five studies reported MT’s positive impact on sensory processing in children with ASD; two studies demonstrated MT’s improvement in patient emotional regulation. Seven studies evaluated the effects of MT on verbal and nonverbal communication skills in children with autism. Analysis revealed that MT significantly improved verbal communication (P = 0.01). A comprehensive analysis highlights the significant role of MT as an intervention that can enhance various functional aspects in children with ASD.

**Figure 8:**
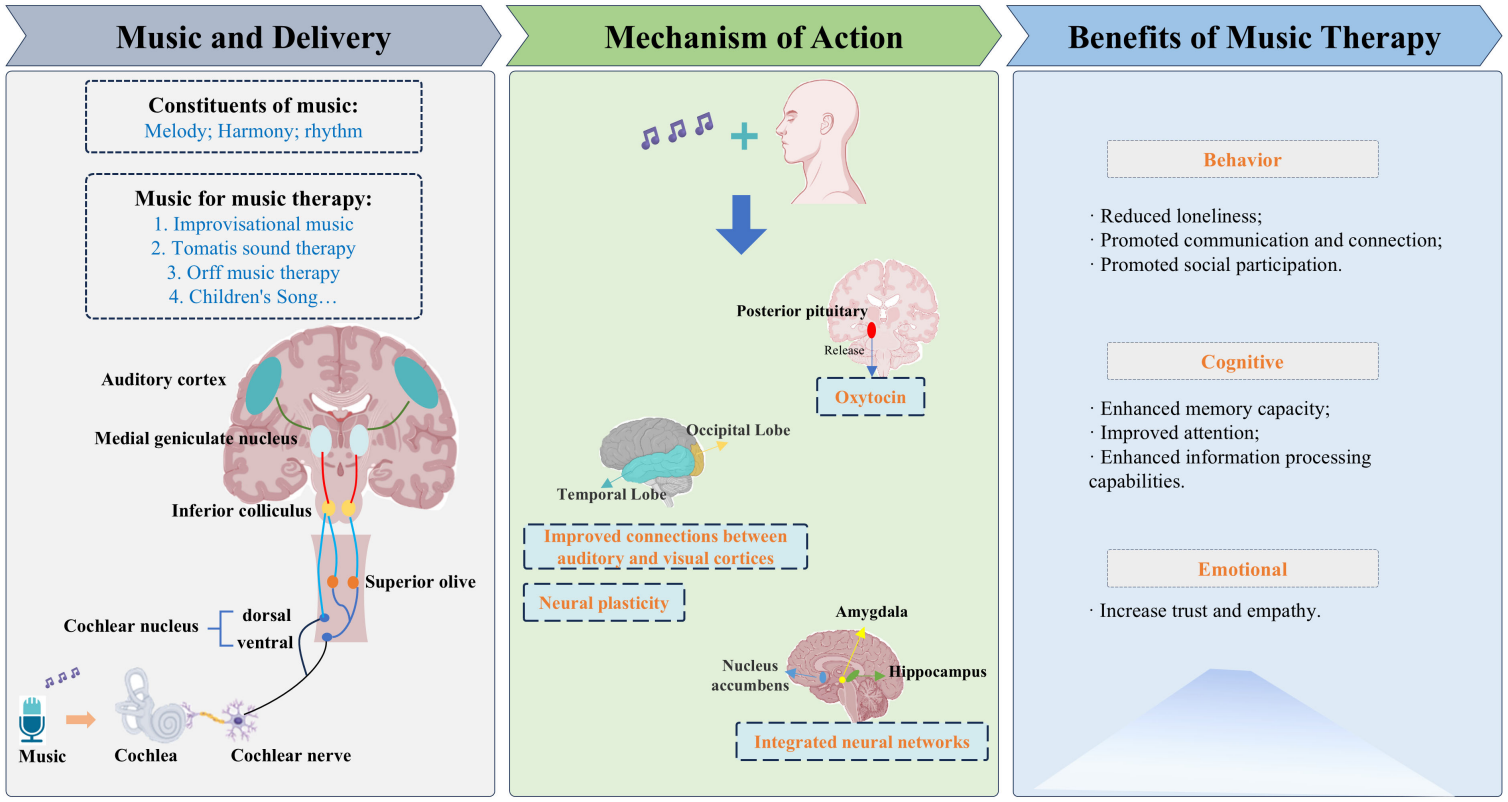
The types of music used for treatment and the mechanisms and benefits of the music in influencing the brain.

The complex mechanisms underlying autism’s multiple overlapping causes still lack clinical treatments targeting its core symptoms. The absence of molecular targets poses a major obstacle to developing new drugs for autism. The following discusses several mechanisms by which MT interventions improve ASD symptoms (**Figure 8**). One of music’s most recognized abilities is its capacity to communicate, induce, and regulate emotional states (39, 40). Music entering the brain is first processed by the auditory mechanisms of our central nervous system. This requires the involvement of neural circuits and other physiological systems that are directly or indirectly connected to the auditory neural circuits responsible for perceiving and processing musical elements (41). Music frequently triggers brain activity in key regions involved in emotional processing, such as the amygdala, hypothalamus, hippocampus, supplementary motor area, cingulate cortex, and frontal cortex (42, 43). The most variable part of the mammalian auditory system is the auditory cortex, specifically the temporal lobe cortex, which receives input from the auditory thalamus and responds to auditory stimuli (44, 45). Research indicated that music can be transmitted through the auditory cortex, repairing damaged areas and enhancing the plasticity of brain neurons (46).

Modern neuroscience methods, such as resting-state functional magnetic resonance imaging, help identify potential beneficiaries more accurately (47). Using magnetic resonance imaging and diffusion tensor imaging probabilistic tractography, impaired thalamocortical connectivity was identified in individuals with autism spectrum disorder (48). Research has found that following MT intervention, individuals with ASD exhibit increased functional connectivity between the bilateral primary auditory cortex and motor cortex regions, score higher on communication measures, and demonstrate reduced hyperconnectivity between auditory and visual association areas (49). Longitudinal fMRI results in healthy older adults revealed that an 8-week receptive music intervention increased functional connectivity from the auditory cortex to reward systems, particularly to the prefrontal cortex (50).

Despite deficits in emotional processing in other domains, individuals with autism are able to successfully identify musical emotions (11, 51). Research demonstrated that auditory stimulation from music can evoke emotional responses, with MT playing a crucial role in activating the peripheral-to-central nervous system pathway within the hypothalamic brainstem autonomic axis (HBA) (52). Pleasant music induces a transient increase in dopamine levels within the nucleus accumbens (NAC) immediately preceding the peak of emotional response (53). Emotional music also activates regions extending to the amygdala and hippocampal dentate gyrus, as well as the thalamus and adjacent midbrain areas associated with arousal and temporal complexity processing (54). Coordinated oscillatory activity between the hippocampus and amygdala is associated with the formation, consolidation, and extinction of episodic and emotional memories (55). Music training not only enhances memory plasticity but also strengthens the ability to retain and process information (56), improved memory also enhances patients’ attention span and language skills (57). Researchers also found that MT intervention enhanced social engagement in children with autism and improved their cognitive abilities (58). Children participating in MT demonstrated greater progress in joint attention and looking at others.

Another mechanism by which MT may reduce behavioral symptoms of ASD involves the release of oxytocin in response to music, This is a neuropeptide released by the posterior pituitary gland. Oxytocin is associated with social bonding, empathy, and trust (59). Research indicated that listening to music is accompanied by an increase in oxytocin levels (60), Oxytocin is believed to enhance social brain function in areas typically identified as underactive in children with autism (61, 62).

Since the early 1950s, MT has been used for patients with ASD (63). Music therapists and children with ASD can employ improvisation or free musical expression as a form of communication. During collaborative music-making, therapists utilize improvisation to facilitate interaction and turn-taking, aiming to leverage this musical relationship to encourage engagement and connection with others (19). Therefore, we believe that a qualified music therapist should possess an outstanding background in music, hold a music therapy certification from the American Music Therapy Association or equivalent national credentials, and have extensive clinical care experience. Additionally, they must be able to promptly adjust treatment plans based on changes in the patient’s emotional state.

The types of music currently used to treat ASD vary, with some employing Orff music, others utilizing Tomatis sound therapy, and still others incorporating improvisational music depending on the musician (**Table 1**), the effects achieved also vary. Because the experience of music is complex, musical cultures vary across different societies, Music affects each person differently (64). Research has found that listening to self-selected music stimulates the auditory and reward systems more effectively than listening to music chosen by researchers (65), Therefore, when implementing MT for individuals with ASD in the future, careful consideration should be given to music selection. Appropriate music should be chosen based on the child’s age and specific background, combining group therapy with personalized approaches.

MT for ASD still faces significant challenges. Music can be divided into three fundamental components (melody, harmony, and rhythm), each connected by overlapping yet distinct neural networks (66, 67). Music is typically combined in multiple ways, resulting in significant heterogeneity during the intervention process (68), This also leads to inconsistent results across different studies. Meanwhile, current research on the effects of MT in ASD populations primarily focuses on children or adolescents, with only a few studies indicating that MT can improve autism symptoms in adults (69). Therefore, more research is needed on adults (70). Although MT interventions can significantly improve certain symptoms in individuals with ASD, with advances in technology and cognitive approaches, an increasing number of studies are beginning to combine music with other intervention methods to enhance symptom improvement outcomes (71, 72).

It is worth noting that this analysis has certain limitations, as some of the included studies were conducted by Chinese researchers. Regional differences and cultural backgrounds may constitute potential confounding factors. Geographic variations also led to differences in the types of music used, which may have contributed to publication bias. In subsequent research, we encourage researchers to conduct and publish studies with broader geographic coverage and involving more diverse participant groups. At the same time, current research primarily reflects short-term efficacy (with the longest intervention period assessed immediately after 6 months). There is a lack of research on the long-term maintenance effects of these interventions, making it impossible to determine whether beneficial effects persist after intervention cessation—a critical piece of information for clinical practice. Therefore, we strongly recommend that future research designs incorporate long-term follow-up surveys (e.g., at 3 months, 6 months, 1 year, or even longer post-intervention) to assess the persistence and decay cycle of MT effects. This will provide essential guidance for determining appropriate intervention cycles.

We also urge researchers to pre-register their clinical trials on internationally recognized public registration platforms prior to initiation, adhere to standardized reporting guidelines, and upload analysis datasets to public data repositories while safeguarding participant privacy and complying with ethical standards. This will significantly enhance the quality, credibility, and utility of research findings.

## Data Availability

The original contributions presented in the study are included in the article/**Supplementary Material**. Further inquiries can be directed to the corresponding author.
