# Supplementary material for: Effectiveness of music therapy for children with autism spectrum disorder: meta-analysis and potential biological mechanisms
Source: Front Psychiatry. 2026 Jan 30;16:1722874. doi: 10.3389/fpsyt.2025.1722874 (PMC12900671; doi:10.3389/fpsyt.2025.1722874)
Supplement: Supplementary file 1 [file Supplementaryfile1.docx]

Supplementary Materials


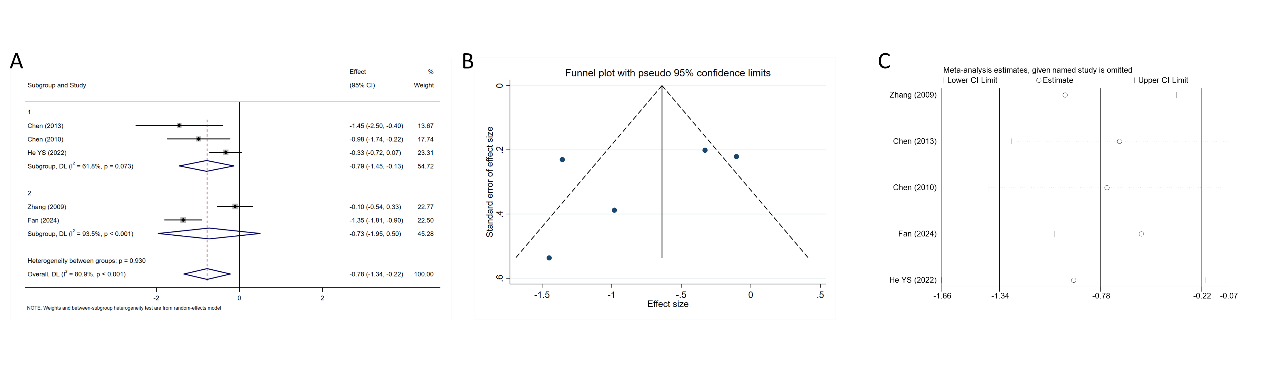


Figure S1. A. Subgroup analysis of ABC total scores; B. Funnel plot of ABC total scores; C. Sensitivity analysis of ABC total scores.


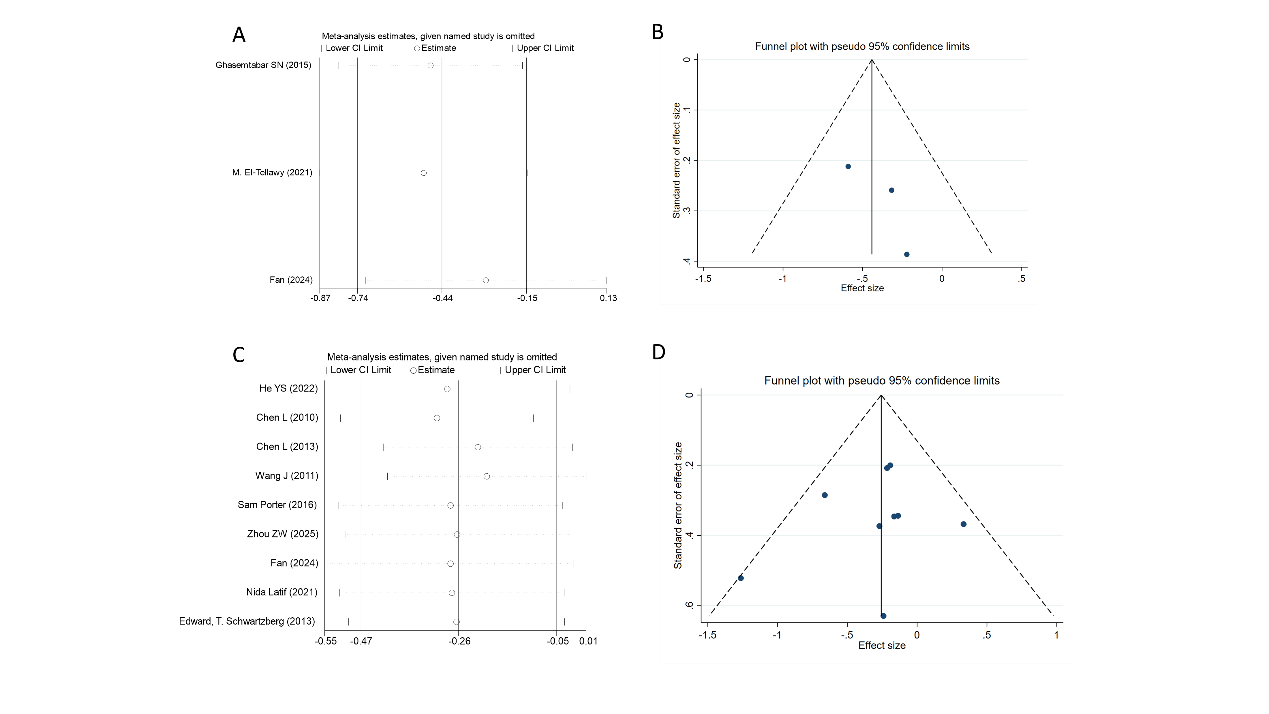


Figure S2. A. Sensitivity analysis of CARs total score; B. Funnel plot of CARs total score; C. Sensitivity analysis of social score; D. Funnel plot of social score.


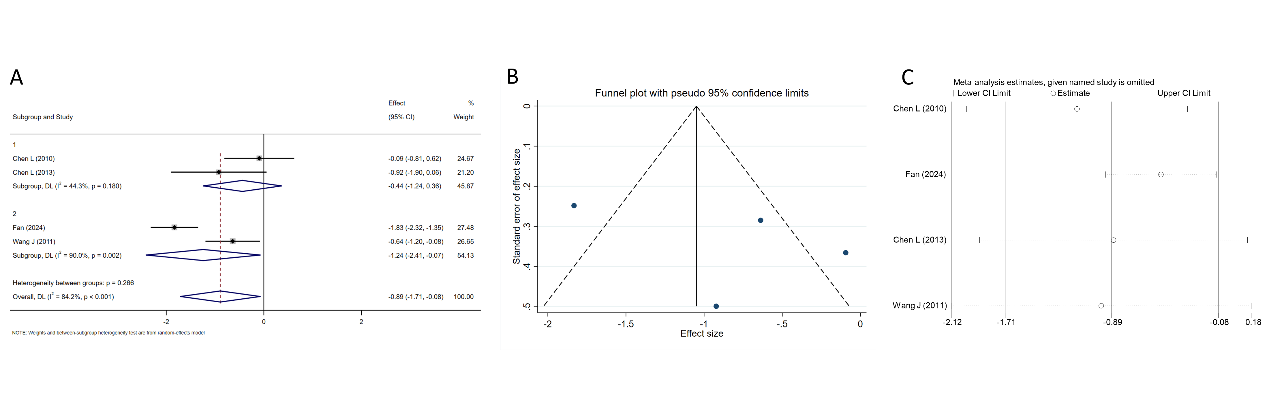


Figure S3. A. Subgroup analysis of sensory scores; B. Funnel plot of sensory scores; C. Sensitivity analysis of sensory scores.


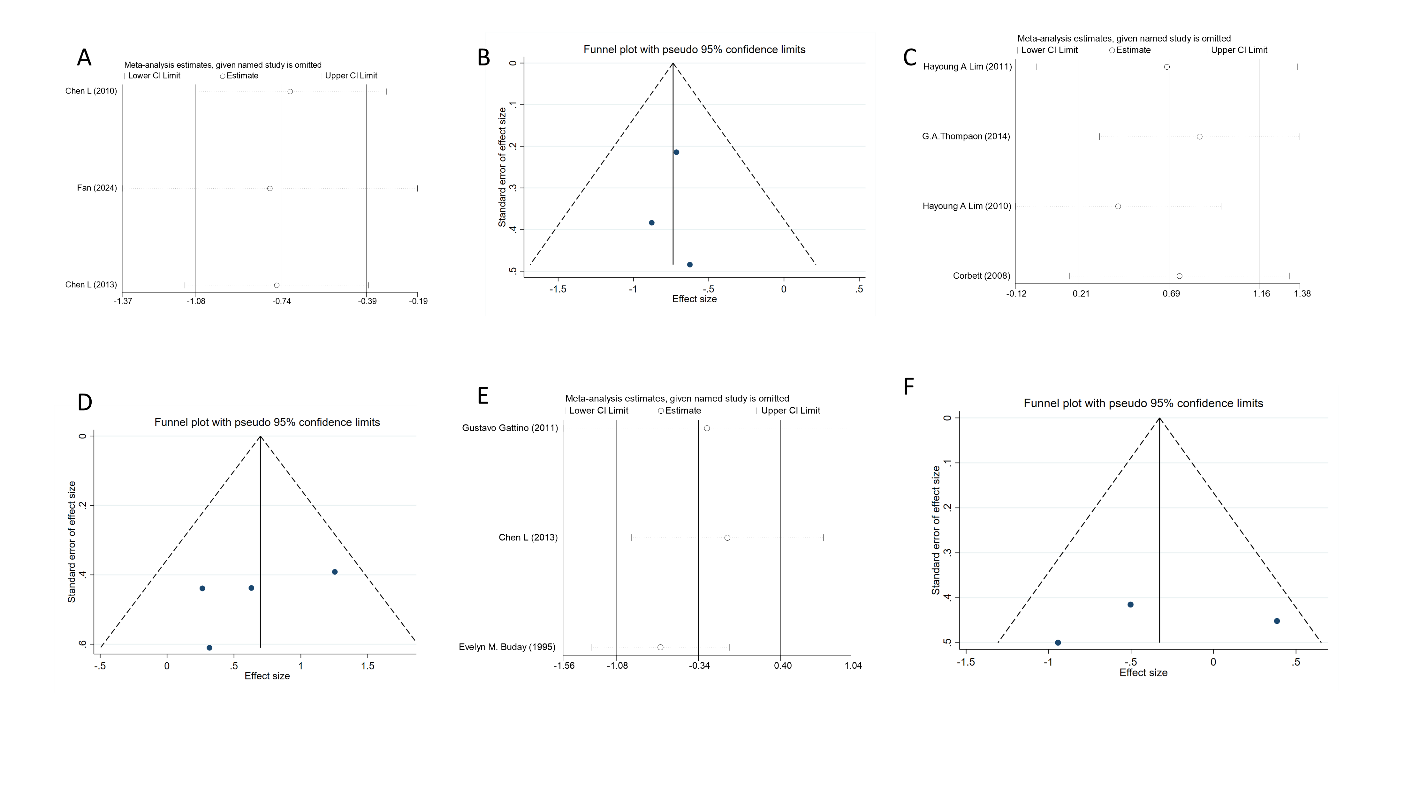


Figure S4. A. Sensitivity analysis of physical scores; B. Funnel plot of physical scores; C. Sensitivity analysis of verbal scores; D. Funnel plot of verbal scores; E. Sensitivity analysis of nonverbal scores; F. Funnel plot of nonverbal scores.
